# Ultrafast optical manipulation of magnetic order in ferromagnetic materials

**DOI:** 10.1186/s40580-020-00246-3

**Published:** 2020-11-10

**Authors:** Chuangtang Wang, Yongmin Liu

**Affiliations:** 1 Department of Electrical and Computer Engineering, Northeastern University, Boston, MA 02115 USA; 2 Department of Mechanical and Industrial Engineering, Northeastern University, Boston, MA 02115 USA

**Keywords:** All-optical manipulation, Magnetization, Ferromagnetic materials, Ultrafast

## Abstract

The interaction between ultrafast lasers and magnetic materials is an appealing topic. It not only involves interesting fundamental questions that remain inconclusive and hence need further investigation, but also has the potential to revolutionize data storage technologies because such an opto-magnetic interaction provides an ultrafast and energy-efficient means to control magnetization. Fruitful progress has been made in this area over the past quarter century. In this paper, we review the state-of-the-art experimental and theoretical studies on magnetization dynamics and switching in ferromagnetic materials that are induced by ultrafast lasers. We start by describing the physical mechanisms of ultrafast demagnetization based on different experimental observations and theoretical methods. Both the spin-flip scattering theory and the superdiffusive spin transport model will be discussed in detail. Then, we will discuss laser-induced torques and resultant magnetization dynamics in ferromagnetic materials. Recent developments of all-optical switching (AOS) of ferromagnetic materials towards ultrafast magnetic storage and memory will also be reviewed, followed by the perspectives on the challenges and future directions in this emerging area.

## Introduction

Applying a magnetic field is the most common method to manipulate magnetization. The magnetization will align along the same direction of the magnetic field since this state has the lowest energy. Other methods based on the effect of electrical currents have been studied. When an electric current passes through a magnetic layer, it will become spin-polarized. The spin-polarized current will exert a spin-transfer torque (STT) on the magnetization. The spin current can also be generated inside a non-magnetic metal due to the spin–orbit coupling (SOC), and the corresponding torque is called spin–orbit torque (SOT). The magnetization switching process in all these approaches is governed by a precessional motion, as described by the thermodynamic Landau–Lifshitz–Gilbert (LLG) equation. The precessional switching is known as the rapid magnetization reversal mechanism in the current magnetic random-access memory (MRAM) devices, driven by a transient magnetic field [1, 2] (~ 2 ps with 1.7 T magnetic field), spin-transfer-torque (sub-200 ps) [3] or spin–orbit torque (400 ps) [4, 5].

There is a demand to control magnetization in a faster and more energy-efficient manner. Using optical pulses to manipulate magnetization is one promising solution. The interaction between ultrafast lasers and magnetic materials has attracted extensive research attention since the discovery of ultrafast demagnetization in ferromagnetic material Ni, which takes place within an unexpected short timescale of 60 fs [6]. It is commonly accepted that a material can be excited to a non-equilibrium state after absorbing the laser energy, where the classic thermodynamics theories are not applicable. Very interestingly, the magnetic states of magnetic materials can be drastically changed and well manipulated during the non-thermodynamic process. Applying pulsed lasers to trigger ultrafast magnetic switching may lead to disruptive MRAM technologies since the material is in a non-equilibrium state and can be reversed at the sub-ps scale [7]. Despite the intense work and significant progress in this field, it is still not very clear where the spin angular moment comes from and how it evolves in an ultrafast manner [8–10].

The AOS of magnetization was found in different materials, including ferrimagnetic dielectrics [11–13], ferrimagnetic metals [14, 15], and ferromagnetic metals [16]. For a dielectric cobalt-substituted garnet, the precessional magnetization switching between two states can be achieved within less than 20 ps using a single linearly polarized pulse [11]. However, the single-crystalline garnet is not compatible with the current complementary metal–oxide–semiconductor (CMOS) technology. On the other hand, efforts to switch the ferrimagnetic magnetic tunnel junction by a single ps-pulse has been proved possible [17]. A good MRAM device requires a large spin polarization for a giant magnetoresistance ratio and a high anisotropy for maintaining thermal stability, especially for a small data bit size. In this context, ferromagnetic materials are more desirable, because they usually have larger anisotropy and spin polarization compared to ferrimagnetic materials. Therefore, it is crucial to understand the behaviors and mechanisms of the ultrafast dynamics and AOS in ferromagnetic metals for future magnetic data storage devices.

In this review, we summarize the recent advances regarding the interactions between ultrafast lasers and ferromagnetic metals. In Sect. 2, important findings of the local and non-local contributions to the ultrafast demagnetization of ferromagnetic metals will be discussed. The laser not only locally excites materials into a non-equilibrium state and promotes the transfer of angular momentum, but also drives hot-electrons to transport, leading to a non-locally demagnetization. In addition to the ultrafast demagnetization, lasers can also induce magnetization dynamics of a magnetic thin film with a locally exerted torques or a nonlocal spin-current driven STT. In Sect. 3, we will discuss the recent experimental and theoretical developments of AOS of ferromagnetic metals. The review concludes in Sect. 4 with a brief outlook for this exciting research field.

## Ultrafast laser-induced magnetization dynamics

### Local ultrafast demagnetization

In the seminal work on the ultrafast demagnetization of Ni [6], Beaurepaire et al. proposed a phenomenological three-temperature model (3TM). In this model, there are three thermalized energy reservoirs: electron, lattice, and spin with the corresponding temperature denoted by *T*_*e*_, *T*_*l*,_ and *T*_*s*_, respectively. The dynamics of the system can be described by three coupled differential equations as follows:

1 $$\begin{gathered} C_{e} \left( {T_{e} } \right)dT_{e} /dt = - G_{el} \left( {T_{e} - T_{l} } \right) - G_{es} \left( {T_{e} - T_{s} } \right) + P\left( t \right) \hfill \\ C_{s} \left( {T_{s} } \right)dT_{s} /dt = - G_{es} \left( {T_{s} - T_{e} } \right) - G_{sl} \left( {T_{s} - T_{l} } \right) \hfill \\ C_{l} \left( {T_{l} } \right)dT_{l} /dt = - G_{el} \left( {T_{l} - T_{e} } \right) - G_{sl} \left( {T_{l} - T_{s} } \right) \hfill \\ \end{gathered}$$

Here *C*_*e*_, *C*_*l*_, and *C*_*s*_ denote the electron, lattice, and spin heat capacity, respectively. *G*_*el*_, *G*_*es*_, *G*_*sl*_ are the electron-lattice, electron-spin, and spin–lattice coupling constant, respectively, and *P(t)* refers to the radiation heating from the incident laser. Upon the impinging of a laser pulse, electrons in a magnetic medium quickly absorb the energy from the laser, and then transfer the energy to spin and lattice reservoirs through the electron-spin and electron-lattice coupling. These interaction constants are set as free variables to fit experimental data. Overall, this model succeeds to describe the behavior of the demagnetization process, while the microscopic origin of demagnetization is neglected.

The Elliott–Yafet (EY) type mechanism is considered as a potential explanation for laser-induced ultrafast demagnetization at the microscopic level [18]. This theory states that because of the spin–orbit coupling, a spin state is an admixture of spin-up and spin-down states, and momentum scattering could couple the two states and cause spin relaxation. The EY type electron–phonon [19, 20], electron–electron [21–23], electron–magnon [24–28], as well as electron-defects scattering [29] have been studied in detail. Koopmans et al. first connected the EY electron–phonon spin-flip scattering to the LLG model by a phenomenological damping term [19]. Based on this mechanism, Koopmans et al. introduced a modified three temperature model, which can be written as [20]

2 $$\begin{gathered} C_{e} \left( {T_{e} } \right)dT_{e} /dt = - G_{el} \left( {T_{e} - T_{l} } \right) + \nabla_{z} \left( {\kappa \nabla_{z} T_{e} } \right) \hfill \\ C_{l} \left( {T_{l} } \right)dT_{l} /dt = - G_{el} \left( {T_{l} - T_{e} } \right) \hfill \\ dm/dt = RmT_{l} /T_{C} \left( {1 - m coth\left( {mT_{C} /T_{e} } \right)} \right) \hfill \\ \end{gathered}$$

**Fig. 1:**
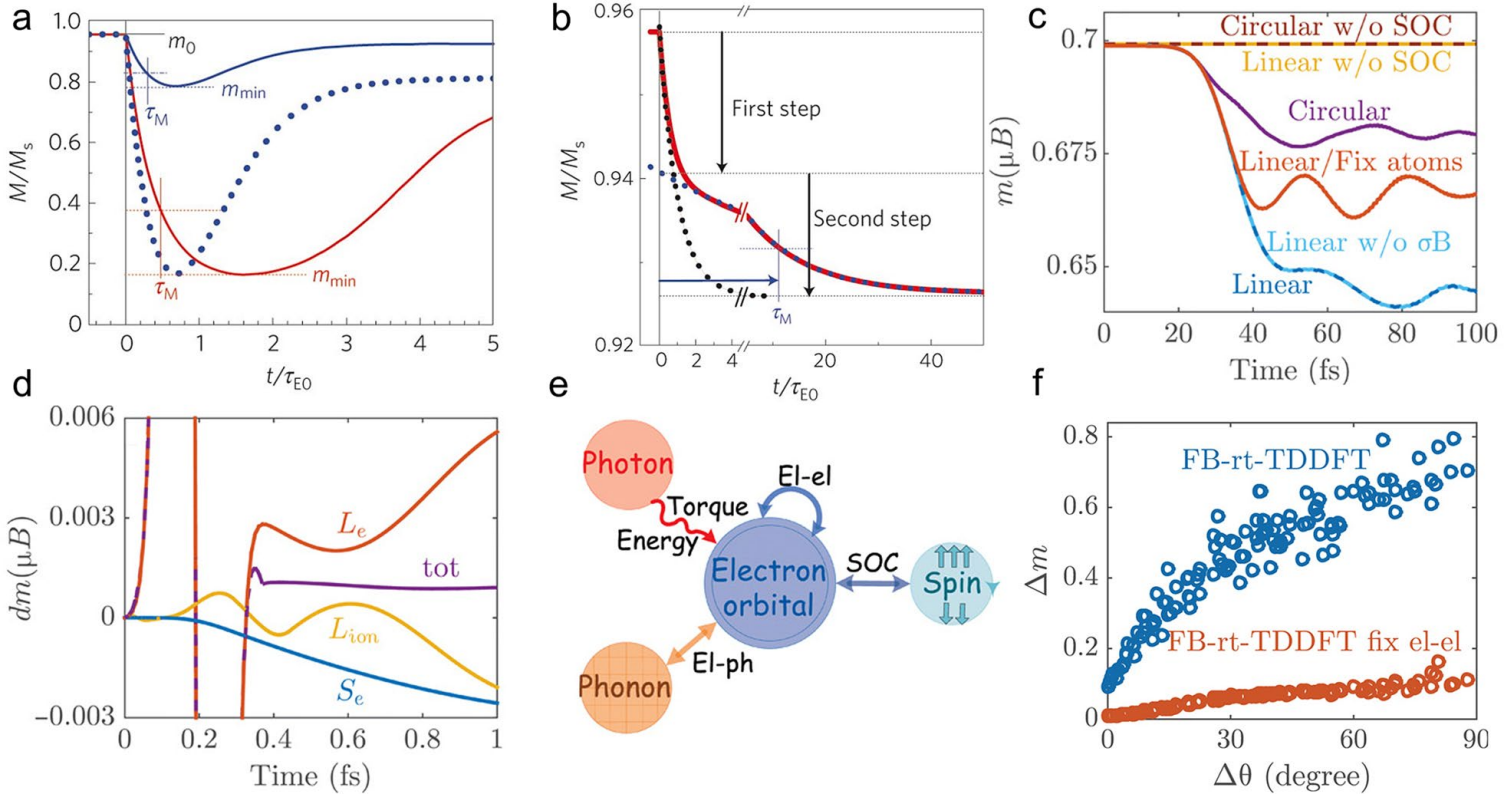
**a** Time evolution of type I demagnetization at low fluence (blue curve), scaled low fluence (blue dotted curve), and high fluence (red curve). **b** Time evolution of type II demagnetization. **c** Time dependence of the magnetic momentum of Ni for different cases. **d** Time evolution of electron spin *S*_*e*_*,* electron orbital *L*_*e*_, ion orbital *L*_*ion,*_ and total angular momentum for a two-atom Ni dimer. **e** Schematic illustration of interactions in the ultrafast demagnetization process. **f** Calculated maximal demagnetization ratio ∆m versus the initial spin disorder ∆θ in Ni. The blue circle and red circle are the fixed basis rt-TDDFT simulation with and without electron–electron interaction, respectively. Figures **a** and **b** are reproduced with permission from ref. [20]. Copyright (2009) Springer Nature. Figure **c**–**f** are reproduced with permission from ref. [45]. Copyright (2019) the authors, some rights reserved; exclusive licensee American Association for the Advancement of Science

Here *κ* is the electronic thermal conductivity, ${T}_{C}$ denotes the Curie temperature, and *m* is the scaled magnetization relative to the magnetization at zero temperature. The factor *R* is a material-dependent scaling factor and proportional to spin-flip probability. In this model, two kinds of dynamics are clarified, namely, type I and type II. For type I dynamics with a large *R* in a typical 3d transition metal, the magnetization dynamics always exhibits a one-step demagnetization feature regardless of the laser fluence, as shown in Fig. 1a. In this regime, the electron-spin equilibration time is shorter than the electron–phonon equilibration time, leading to the remagnetization where the angular momentum is transferred from spins to phonons. However, in the case of type II with small *R* (like Gd), the electron-spin equilibration establishes much more slowly than the electron–phonon equilibration, which means phonons transfer angular momentum to spins after equilibrating with electrons, contributing to a two-step demagnetization process (Fig. 1b). To further improve the model, people have considered the direct spin-phonon coupling term and the spin system heat capacity [30]. In the meantime, there are some arguments that the ultrafast demagnetization does not extensively depend on the EY type electron–phonon interaction [31, 32].

The electron–electron scattering is naturally considered in the process of electron thermalization. It is shown that an EY type demagnetization based on electron–electron scattering rather than electron–phonon could also be a potential mechanism [21]. In the presence of SOT, the optically excited spin-polarized electrons undergo the inter-band Coulomb scattering, and then redistribute between majority and minority bands, leading to the demagnetization. In the work of Mueller et al*.* [22, 23], the authors claimed that both EY type electron–electron and electron–phonon scatterings could contribute to the ultrafast demagnetization process. On the other hand, unlike the case of electron–electron scattering, the SOT prompts the transfer of spin angular momentum to orbital angular momentum via electron–magnon scattering [24, 27]. By simultaneously extracting time-resolved reflectivity and magneto-optical Kerr signals, Carpene et al*.* extrapolated the electron–phonon relaxation time, which is about 240 fs in comparison with the characteristic demagnetization time of 50 ~ 75 fs [24]. Therefore, the electron–magnon spin-flip scattering was proposed to be responsible for the magnetization reduction. However, the later X-ray magnetic circular dichroism measurement did not find the corresponding result supporting momentum transfer [25]. Overall, the contribution from the electron-magnon scattering was found too small to explain experimental results [27].

Density function theory (DFT) [33], atomistic spin dynamics (ASD) model [34], and micromagnetic (macroscopic) simulations [35] are the three most used methods to model ultrafast magnetism dynamics. The localization of magnetic moment to atomic sites is the physical basis of the ASD model. The atomic spin dynamics is governed by Langevin dynamics and corresponding stochastic LLG equation. Although ASD assumes a constant magnetization length at different temperatures, it reproduces experimental results very well thanks to its atomistic spin size [36, 37]. However, ASD is limited to the nanometer length scale because of the small unit spin size (several angstroms). Therefore, the macroscale Landau–Lifshitz–Bloch (LLB) model is more suitable for laser-induced magnetization dynamics [38–40]. LLB model is derived based on ASD by substituting many atomistic spins into a larger unit cell. It contains both longitudinal and transverse damping terms, while the LLG equation only has a transverse one. Moreover, the magnetic parameters of LLB are temperature dependent and the connection between EY type spin-scattering and LLB roots in the LLB damping terms [39]. It is expected that a multiscale method combining the three magnetism simulations would produce more accurate results [41]. Nevertheless, neither ASD nor LLB simulation contains the information of the flow of angular momentum, despite the presence of a thermal noise term representing the source of angular momentum.

The aforementioned models use certain assumptions, and take a parameterization approach to fit experiments [10, 42]. In contrast, a fully ab initio real-time time-dependent density functional theory (rt-TDDFT) without requiring any empirical parameters is highly desired to describe the ultrafast demagnetization [43, 44]. In a recent work, Chen and Wang applied rt-TDDFT to study ultrafast demagnetization mechanisms [45]. Using a new algorithm that is fast and capable of simulating a large domain, the authors first investigated the ultrafast demagnetization in bulk ferromagnetic Ni exposed by a laser pulse with a duration of 60 fs, considering the role of phonon, light polarization, SOC, and Zeeman interaction. The results are shown in Fig. 1c. When atoms are fixed, it is observed that phonons are frozen and demagnetization is reduced by 40%, indicating the role of phonons. When changing the polarization from the linear to circular, the demagnetization is reduced because light absorption is different. To study the effect of direct light-spin interaction, the SOC is turned off while the Zeeman term *σ·B* is kept. One can see that neither linear nor circular polarization without SOC will induce demagnetization. While keeping SOC but turning off Zeeman interaction, there is no observable difference, indicating the direct light-spin interaction is negligible. With the above discussions, one can conclude that the light-orbital interaction and SOC are responsible for the demagnetization. The next vital question is how angular momentum flows between different angular momentum reservoirs. The time evolution of electron spin, electron orbital, ion orbital, and total angular momentum are plotted in Fig. 1d. The orbital angular momentum changes significantly within the pulse duration. Both spin (blue curve) and ionic (yellow curve) angular momentum slightly but fast vary, resulting from the SOC and electron-nuclear Coulomb interaction, respectively. The overall picture of angular momentum transfer is schematically shown in Fig. 1e. Nevertheless, the simulated demagnetization is only 8.3% (see Fig. 1c in ref. [11]), much smaller than experimentally observed 50% demagnetization under the same laser fluence [1, 46]. It turns out that the initial thermal spin disorder [47] at room temperature plays a critical role in ultrafast demagnetization. Figure 1f shows the representative results, where the initial disorder *∆θ* remarkably alters the demagnetization rate. The zero disorder only leads to demagnetization of about 10% while the system reaches 40% demagnetization at *∆θ* = 22.3% corresponding to room temperature. Earlier studies also manifest the influence of thermal spin disorder in the demagnetization process [19, 48]. In the meantime, the electron–electron interaction, which is not considered in many analytical explanations mentioned above, has also been investigated by turning off the many-electron correlation effect. One can see from Fig. 1f that there is only less than 10% demanganization even with the high spin disorder. Overall, the laser excites the electron orbital. Due to the SOC, such an excited electron orbital state torques the electron spin. The initial spin disorder substantially strengthens the torque because of its randomness, leading to collective ultrafast demagnetization.

### Non-local ultrafast demagnetization

**Fig. 2:**
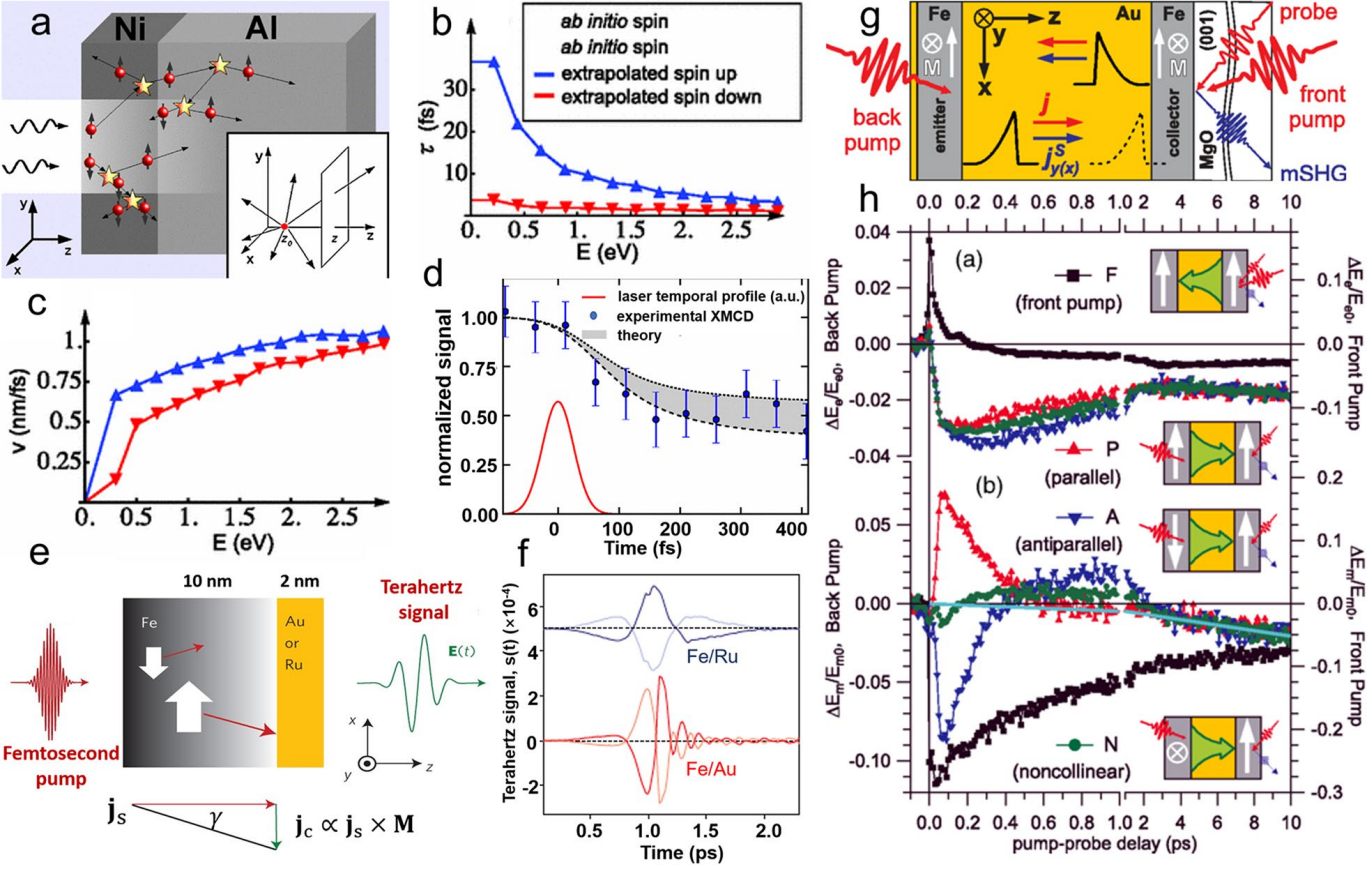
**a** Schematic of the laser-excited superdiffusive processes. **b** Lifetimes and **c** velocities of electrons in Ni. **d** Calculated demagnetization of Ni, in comparison with X-ray magnetic circular dichroism data. **e** Schematic illustration of Terahertz emission after the laser excitation. The electrical current j_c_ is generated by the inverse spin Hall effect from the spin current j_s_. **f** Terahertz emission by laser pumping Fe/Ru and Fe/Au thin films. The signal inverts when the magnetization inverts (dark to light curves). **g** Schematic of the pump-probe approach. **h** Odd and even part of the time-dependent SHG signal. Figure **a** and **d** are reproduced with permission from ref. [50]. Copyright (2010) American Physical Society. Figure **b** and **c** are reproduced with permission from ref. [51]. Copyright (2012) American Physical Society. Figure **e** and **f** are reproduced with permission from ref. [53]. Copyright (2013) Springer Nature. Figure **g** and **h** are reproduced with permission from ref. [58]. Copyright (2017) American Physical Society

Apart from the local spin scattering mechanisms, non-local hot-electron transport of spin angular momentum can also contribute to ultrafast demagnetization. In 2008, Malinowski et al*.* experimentally demonstrated that the spin-polarized hot-carrier transport between two Co/Pt layers could enhance ultrafast demagnetization in Co/Pt/Ru/Co/Pt multilayer [49]. A semiclassical superdiffusive spin transport model was proposed afterward by Battiato et al*.* to explain the demagnetization of metallic ferromagnets [50, 51] (Fig. 2a). In the ultrafast demagnetization case, the excited electrons from the *d* band to the *sp*-like band are non-equilibrated, which cannot be described by the standard diffusion or ballistic diffusion model. Instead, spin and energy-dependent parameters, such as electron velocities and lifetimes, are adopted to simulate the band structure behavior. The energy-dependent electron velocities and lifetimes in Ni are shown in Fig. 2b and c. In the heterostructure consisting of ferromagnetic Ni and non-magnetic metal Al, the longer lifetime of spin-majority enables it to pass the Ni/Al interface and enter the non-magnetic layer, while the spin-minority remains in the ferromagnet layer, leading to the demagnetization of Ni. The computed time evolution of magnetization in Ni is consistent with the experimental pump-probe X-ray magnetic circular dichroism results, as shown in Fig. 2d. The demagnetization ratio reaches 50% within 200 fs after the excitation of a 100-fs pump laser. However, in a later experimental study, the spin current contribution was found to play a negligible role [52]. The time-resolved magneto-optical Kerr effect (TR-MOKE) signals collected from the front and back side of illumination in Ni/MgO and Ni/Al are identical, suggesting that the spin current contribution is negligible, or its contribution is much smaller than the local spin-scattering contribution. Nevertheless, the laser-induced spin current has been identified in many studies. In the following, we will summarize recent works on laser-induced spin current and its role in ultrafast demagnetization based on different characterization techniques.

It is difficult to unambiguously unveil spin current contribution to the ultrafast demagnetization solely by visible or near-infrared TR-MOKE. Additional methods have been used to identify spin current, including terahertz spectroscopy [53–56], optical second harmonic generation (SHG) spectroscopy [57–60], X-ray or extreme-ultraviolet (XUV) spectroscopy [61–63], and time-resolved complex Kerr spectroscopy [64–67]. Terahertz spectroscopy studies of Fe/Au and Fe/Ru heterostructure are presented in Fig. 2e [53]. An in-plane magnetic field is applied along the *y*-direction to saturate the magnetic film. Based on the inverse spin Hall effect, the pumped longitudinal spin current *J*_*s*_ is converted to a transverse charge current *J*_*c*_. The relation between the two currents is given by

3 $${\varvec{J}}_{{\varvec{c}}} = {\upgamma }{\varvec{J}}_{{\varvec{S}}} \times {\varvec{M}}/\left| {\varvec{M}} \right|$$

where γ denotes the spin Hall angle. One can see from Fig. 2f that the generated terahertz signal emerges and attenuates within 1 ps in Fe/Ru and Fe/Au. Furthermore, the signal sign reverses when an opposite magnetic field is applied, showing the characteristic of the spin current. On the other hand, the spin current inside the metallic film can break its inversion symmetry, generating SHG signals [68]. In a Fe/Au/Fe (100) heterostructure, the spin current flows from the Fe layer to the Au layer after excitation, as shown in Fig. 2g [57, 58]. Both the bulk dipole current and spin current will induce a second-harmonic field ${E}_{e}^{C}\propto {v}_{z}$ and ${E}_{m}^{SC}\propto {v}_{z}{s}_{y}$, respectively. ${v}_{z}$ is the electron velocity, which is independent of magnetization; ${s}_{y}$ is the spin polarization, which depends on magnetization. Figure 2h plots the time-resolved odd and even part of the SHG signal. By changing the pump direction, one can reverse ${v}_{z}$, resulting in opposite odd and even signals. However, one only observes reversing odd part when the magnetization is changed.

**Fig. 3:**
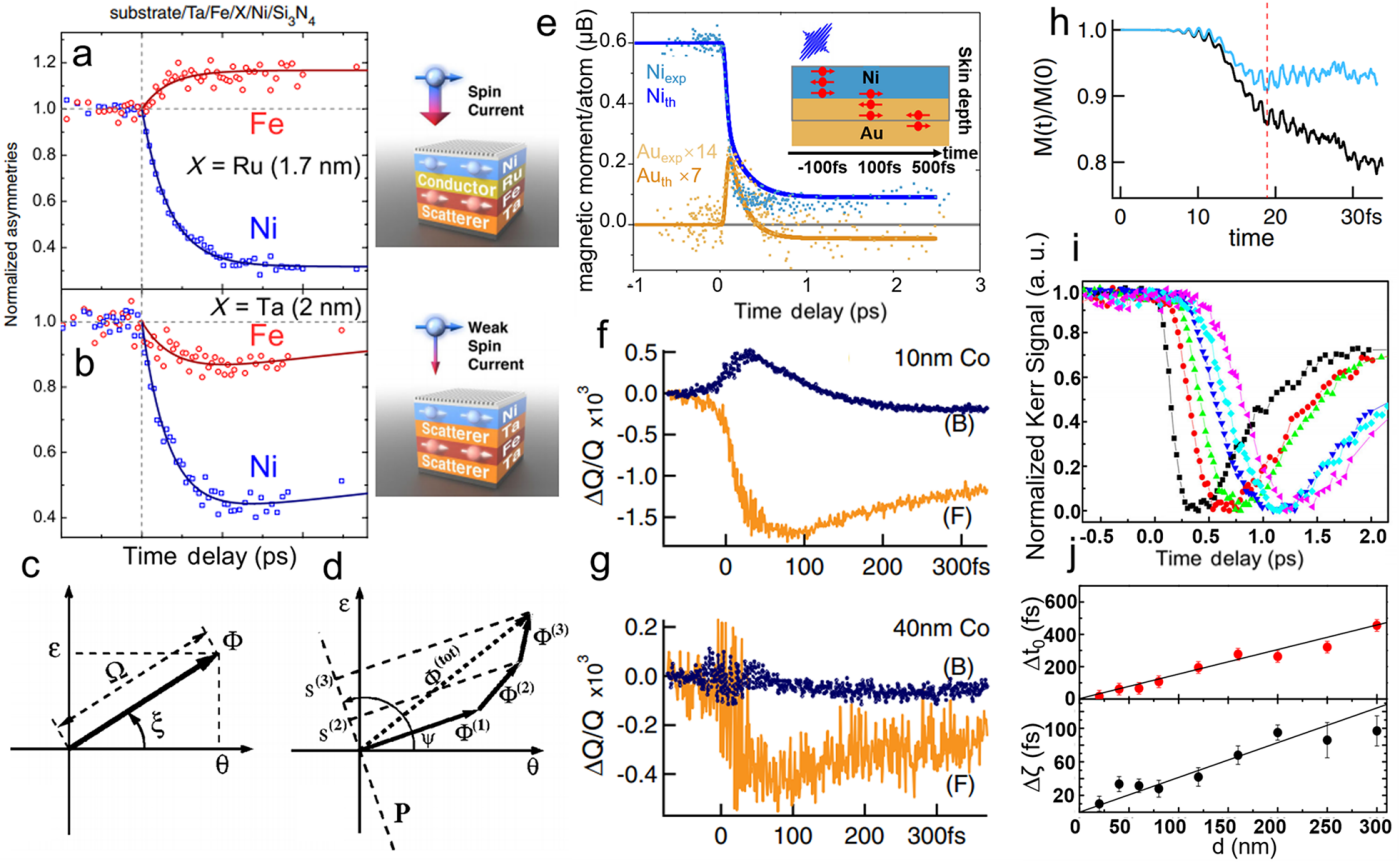
Magnetization dynamics of Fe and Ni layer in **a** Fe/Ru/Ni and **b** Fe/Ta/Ni multilayers. **c** Kerr vector $\Phi$ in the complex plane. **d** Representation of relation that the measured Kerr signal ${S}^{(i)}$ is the projection of Kerr vector ${\Phi }^{(i)}$. **e** Kerr signal measured at a different detection angle corresponding to Ni or Au layer. Magnetization dynamics of **f** 10 nm and **g** 40 nm Co films on their back (B) and front (F) sides. **h** Calculated ultrafast demagnetization for the Co film with the contribution of both spin flips and spin transport (black line) or only spin transport (blue line). **i** Time-dependent magneto-optical Kerr signal measured at different Cu thickness. **j** The time delay of the onset of demagnetization (top) and the characteristic demagnetization time (bottom) as a function of Cu thickness. Figure **a** and **b** are reproduced with permission from ref. [62]. Copyright (2010) American Physical Society. Figure **c** and **d** are reproduced with permission from ref. [71]. Copyright (2002) American Physical Society. Figure **e** are reproduced with permission from ref. [66]. Copyright (2017) American Physical Society. Figure **f**, **g**, and **h** are reproduced with permission from ref. [67]. Copyright (2017) American Physical Society. Figure **i** and **j** are reproduced with permission from ref. [75]. Copyright (2016) American Physical Society

X-ray and XUV are suitable to characterize element-specific magnetization dynamics because the probe beam has high energy [69]. To study the spin current transport, ferromagnetic Fe and Ni layers are stacked with different thin insertion layers of Ru, Ta, W, or insulating Si_3_N_4_ [61, 62]. Two representative results are presented in Fig. 3a and b. The XUV transverse magneto-Kerr effect reveals that there is a 68% reduction of Ni magnetization and surprisingly a 16% increment in Fe with a 1.7 nm insertion layer of Ru. However, both magnetization of Fe and Ni decreases with a 2 nm insertion layer of W. The spin-flip scattering theory cannot explain the observed abnormal increase of magnetic moment in Fe. The spin-majority preferentially flows from Ni to Fe. The difference between two heterostructures arises from the longer spin diffusion length of Ru (14 nm) than that of W (4.8 nm), and hence larger spin momentum transfer in the case of Ru [70].

SHG detection and X-ray source require a great expense or large facilities. Taking advantage of the in-depth sensitivity of the complex magneto-Kerr effect, we could use a standard TR-MOKE setup to characterize spin current transport [71]. The complex Kerr vector $\Phi =\theta +i\epsilon =\Omega {e}^{i\xi }$ is represented in the complex plane and the total Kerr response ${\Phi }_{total}$ is the vector summation of different layers ${\Phi }_{total}={\Phi }_{1}+{\Phi }_{2}+{\Phi }_{3}$, as illustrated in Fig. 3c and d. By controlling the projection axis orthogonal to ${\Phi }_{1}$, we can measure the projected Kerr signal from ${\Phi }_{2}+{\Phi }_{3}$. In this way, one could detect the magnetization dynamics of a specific layer [64–66]. One example is shown in Fig. 3e. The Ni layer has an 87% ultrafast demagnetization and the Au layer has a corresponding shape of magnetic response [66], suggesting that the superdiffusive spin current flows from Ni to Au. Shokeen et al*.* employed extremely narrow 10 fs optical pulses to study the magnetization dynamics in a single layer of Ni and Co layer [67]. This is different from the previous case in which no evidence of superdiffusive spin current effect was found by using a 75-fs laser [52]. The experimental temporal Voigt modulus Q signals probed on the back and the front side of the sample in Co thin film are plotted in Fig. 3f and g, respectively. The Voigt modulus is proportional to the magnetization. For the 10 nm Co layer, $\Delta \mathrm{Q}/\mathrm{Q}$ on the back side shows a reversed sign during the first 50 fs compared to the front side, implying that a significant volume of majority spins propagates towards the back side. Nevertheless, only neglectable $\Delta \mathrm{Q}/\mathrm{Q}$ is observed in the 40 nm Co film, indicating that the spin current is scattered during transportation. In terms of simulations, the previously discussed superdiffusive model [50, 51] neglects SOC, so that only spin current contribution matters in demagnetization. In this work, a fully ab initio approach with time-dependent DFT is performed. The calculated magnetization dynamics with and without SOC is presented in Fig. 3h. Two curves are similar only in the first 20 fs, indicating that spin-carriers diffusion contributes significantly while spin flips dominate after the first 20 fs.

The aforementioned spin-polarized hot electrons are generated in a ferromagnetic layer after the laser excitation and then flows out of the exciting area, leaving it demagnetized. One related question is how non-polarized hot electrons affect the demagnetization process. In 2013, Eschenlohr et al*.* reported a slight 70 fs onset delay of demagnetization in an Au/Ni bilayer structure compared to a bare Ni sample, which suggests a transport behavior [72, 73]. This non-polarized hot-electron contribution was also observed in subsequent works [74–76]. To eliminate direct optical excitation while keeping hot electron transport, a thick Cu was used for its exceptionally long mean free path of hot electrons [77]. In Fig. 3i, the Kerr signal is delayed when the Cu thickness increases [75]. Both the onset time delay of demagnetization and characteristic demagnetization time is fitted to be linearly proportional to Cu thickness (Fig. 3j). The linear evolution of the delay time and estimated high ballistic velocity (0.68 × 10^6^ m/s) can only be ascribed to hot electrons. Besides, the broadening of the hot electron pulse gives rise to the observed linear variation of demagnetization time as a function of Cu thickness.

### Ultrafast laser-induced torque

In this section, we will review some experimental works on manipulating magnetization dynamics using laser-induced torques. There are several demonstrated laser-induced torques exerting on metallic ferromagnetic materials, such as magnetic anisotropy change-induced torque [78, 79], inverse Faraday effect (IFE) induced torque [80–83], optical spin-transfer torque (OSTT) [80–82], and STT [84, 85]. All of them take place within a timescale faster than spin precession. Therefore, the magnetization dynamics is governed by a transient tilt of magnetization from the effective magnetic field and successive spin precession around the effective magnetic field. For example, in the case of the magnetic anisotropy change-induced torque, laser energy would thermalize metallic ferromagnets and reduce the magnetization amplitude [78, 79], or change the exchange coupling strength in composite thin films [86, 87]. Both effects will change the effective magnetic field direction and produce torque.

When circularly polarized light interacts with metallic ferromagnets, the optical selection rules generate spin-polarized carriers which then couple with magnetic moment **M**. This is called OSTT [88]. Another helicity-driven spin torque is the IFE induced torque, which was originally demonstrated in insulated and metallic ferrimagnets [89, 90]. The magnetization dynamics induced by the two kinds of torques can be described by the following equation [81]:

4 $$\frac{{d{\varvec{M}}}}{dt} = - \frac{1}{M}{\varvec{M}} \times \left( {{\varvec{M}} \times \frac{{d{\varvec{m}}_{sp} }}{dt}} \right) - \gamma {\varvec{M}} \times {\varvec{B}}_{op}$$

**Fig. 4:**
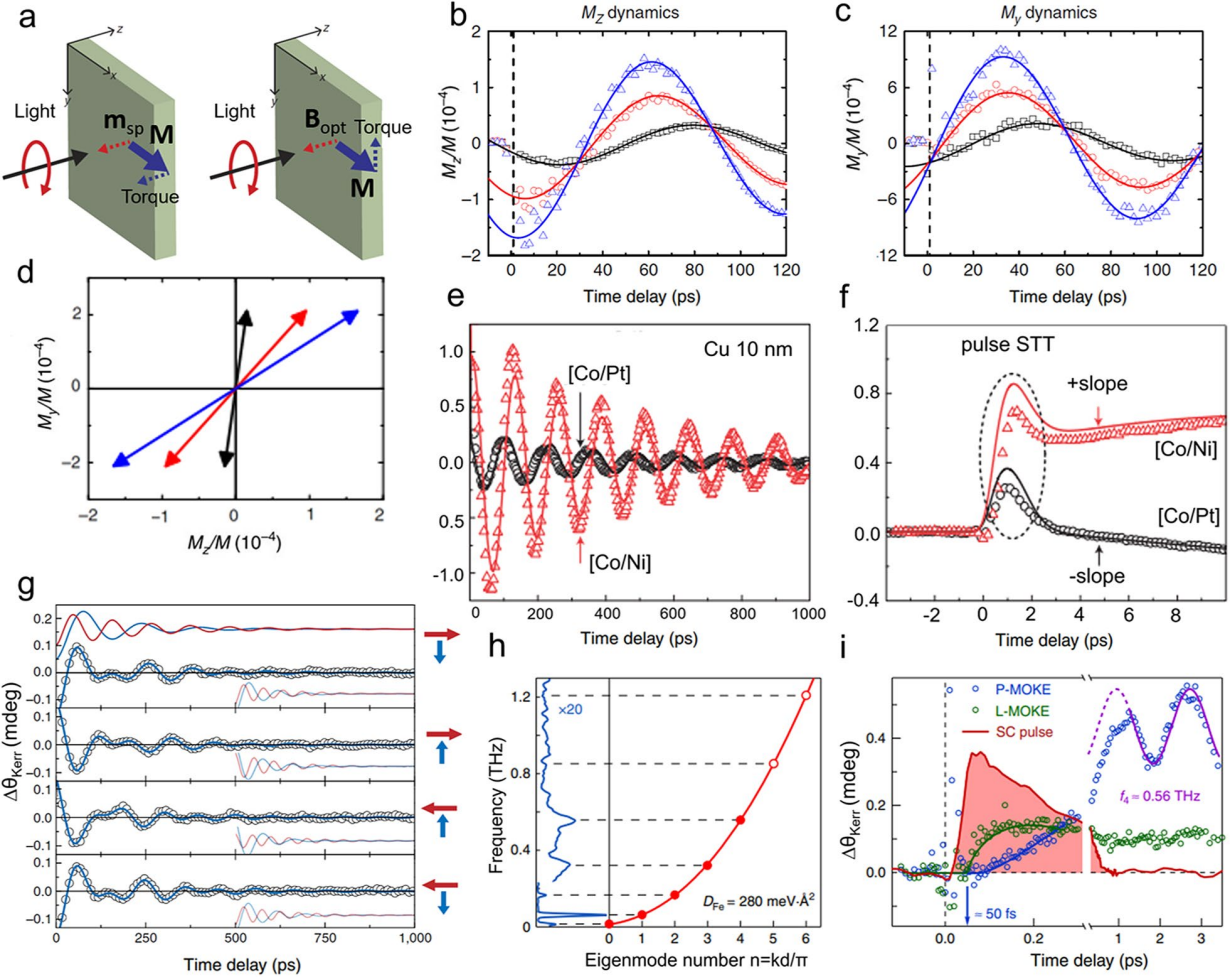
**a** Schematic representation of OSTT and IFE torques. Helicity-dependent **b** *M*_*z*_ and **c** *M*_*y*_ magnetization dynamics of Co (10) thin films with capping layer of 2 nm Au (black curve), 2 nm Pt (red curve), and 4 nm Pt (blue curve). **d** Representation of helicity-dependent *M*_*z*_ and *M*_*y*_ tilting at 1 ps of Co (10)/capping. **e** Magnetization dynamics of [Co/Pt] (or [Co/Ni])/Cu/CoFeB heterostructure. **f** Magnetization dynamics of [Co/Pt] (or [Co/Ni])/Cu/CoFeB heterostructure at finer timescales from − 4 to 10 ps. **g** Time-resolved polar magneto-optical Kerr signal of the bilayer of four configurations. **h** Fourier spectrum of the magnetization dynamics (left) and calculated spin waves dispersion (right). **i** TR-MOKE signal at ultrashort timescales. Figure **a**, **b, c**, and **d** are reproduced with permission from ref. [81]. Copyright (2017) the Authors. Figure **e** and **f** are reproduced with permission from ref. [92]. Copyright (2015) Springer Nature. Figure **g** is reproduced with permission from ref. [84]. Copyright (2014) Springer Nature. Figure **h** and **i** are reproduced with permission from ref. [85], under a Creative Commons Attribution 4.0 International License

Here ***M*** and *M* are the unit vector and magnitude of magnetization of the ferromagnet, respectively, and *γ* is the gyromagnetic ratio. ***m***_*sp*_ denotes the OSTT-induced spin polarization and ***B***_*opt*_ is the IFE related effective magnetic field along the light propagation direction. In the experimental design of Choi et al. (Fig. 4a), the resulting torques from ***m***_*sp*_ and ***B***_*opt*_ are along the *z*- and *y*-direction, respectively. Due to the transient torques, the spin will precess in the *y*–*z* plane with respect to the effective magnetic field along the *x*-direction. The extracted dynamics of helicity-dependent (HD) *M*_*z*_ and *M*_*y*_ dynamics of 10 nm Co thin films capped by 2 nm Au, 2 nm Pt, and 4 nm Pt are plotted in Fig. 4b and c. The phase delay φ of *M*_*z*_ and *M*_*y*_ are obtained by fitting the data with a damped cosine function $\mathit{cos}\left(2\pi ft-\varphi \right)exp(-t/\tau )$, which represents the initial tilt of magnetization. The initial helicity-driven *M*_*z*_ and *M*_*y*_ tilting are graphed in Fig. 4d for better illustration. Clearly, the *M*_*y*_ tilting is the same for all three capping layers while the *M*_*z*_ tilting increases from Au case to Pt case. Treating IFE as a transient magnetic field thus will not contribute to spin polarization. Therefore, the authors conclude that the IFE dominates inside the ferromagnetic layer while OSTT is induced by the capping layer.

In addition to OSTT-driven spin polarization, superdiffusive spin-polarized carriers generated by demagnetization [84, 91, 92], thermal [92] and non-thermal [85] spin Seebeck effect can also influence the magnetization dynamics. Choi et al*.* studied a stack of out-of-plane-magnetized [Co/Pt] or [Co/Ni], separation Cu layer, and orthogonal in-plane-magnetized CoFeB [92]. After pumping [Co/Pt] or [Co/Ni] multilayers with an ultrafast pulse laser, spin current that are driven by demagnization and spin dependent Seebeck effect (SDSE) would diffuse through Cu and accumulate in CoFeB layer, and then exert a torque. From Fig. 4e, we can see that the precession amplitude of CoFeB with [Co/Ni] is five times larger than the counterpart with [Co/Pt]. However, the observed demagnetization-induced spin accumulation is only 2 times larger [see Fig. 3 in ref. [93]], implying that the SDSE contribution is dominant. Moreover, there is a positive slope of magnetization dynamics for [Co/Ni] and a negative slope for [Co/Pt] after 3 ps as shown in Fig. 4f. This result reveals the distinct signs of SDSE-driven torque because of the different spin relaxation time between [Co/Ni] and [Co/Pt]. Since the heat current lasts much longer than pulse demagnetization-driven current, the SDSE-driven STT is considered to be significant. The demagnetization-driven spin current transfer was also studied with a typical TR-MOKE measurement, which reflects a voluminal Kerr signal contribution. In the work carried out by Schellekens et al*.*, an in-plane magnetized thin Co layer servers as a spin emitter and a perpendicular magnetized [Co/Ni] multilayer is a spin collector [84]. With femtosecond laser pumping, the demagnetization-driven spin current diffuses from Co/Cu interface to [Co/Ni] layer. Four configurations have been studied as shown in Fig. 4g. During the measurements, an external in-plane magnetic field is applied, so that the spin precession will naturally be excited in [Co/Ni] by the laser-induced anisotropy change but not in Co. TR-MOKE reveals a mixed spin precession behavior. The fitting results are shown in Fig. 4g. There are two precessional modes, the low-frequency and the high-frequency mode originating from [Co/Ni] and Co layer, respectively. Interestingly, two precessional phases are consistent with each other and dependent on the [Co/Ni] layer, implying the existence of STT. The result also shows that STT could excite a high-frequency precession mode. STT has been further investigated in an epitaxial grown Fe/Au/Fe/MgO (001) heterostructure [85]. Unlike the thermal case in which carriers with energy ≤ 100 meV around Fermi layer transport [93], the non-thermal Spin-dependent Seebeck effect drives excited electrons in Fe layer to pass through the Fe/Au interface. The spin-polarized electrons are then absorbed by the collector Fe layer. The complex TR-MOKE setup measures the magnetization dynamics in the collector layer, which shows multiple oscillating frequencies. Based on Fourier analysis, the result manifests five standing spin-wave modes with the highest frequency up to 0.56 THz (Fig. 4h). The initial stage of magnetization dynamics can be seen in Fig. 4i, where the start of magnetization dynamics is delayed by 50 fs, suggesting the spin transport characteristic.

## All-optical switching of ferromagnetic materials and nanostructures

One big step towards ultrafast, energy-efficient magnetic storage was the demonstration of all-optical helicity-dependent switching (AO-HDS) of ferrimagnetic GdFeCo thin films in 2007 by using a single femtosecond laser pulse [14]. It was shown that magnetization can be deterministically controlled by left- and right-circularly polarized light. Since then, helicity-independent (HI) switching has been found in different ferrimagnetic metallic alloys. One generally accepted mechanism of this phenomenon is transient ferromagnetic state within sublattices induced by laser heating [15, 94], whereas it cannot be applied to later discovered all-optical switching in ferromagnetic materials with a single lattice [16]. Extensive work has been conducted to better understand and realize all-optical switching in ferromagnetic materials and nanostructures.

**Fig. 5:**
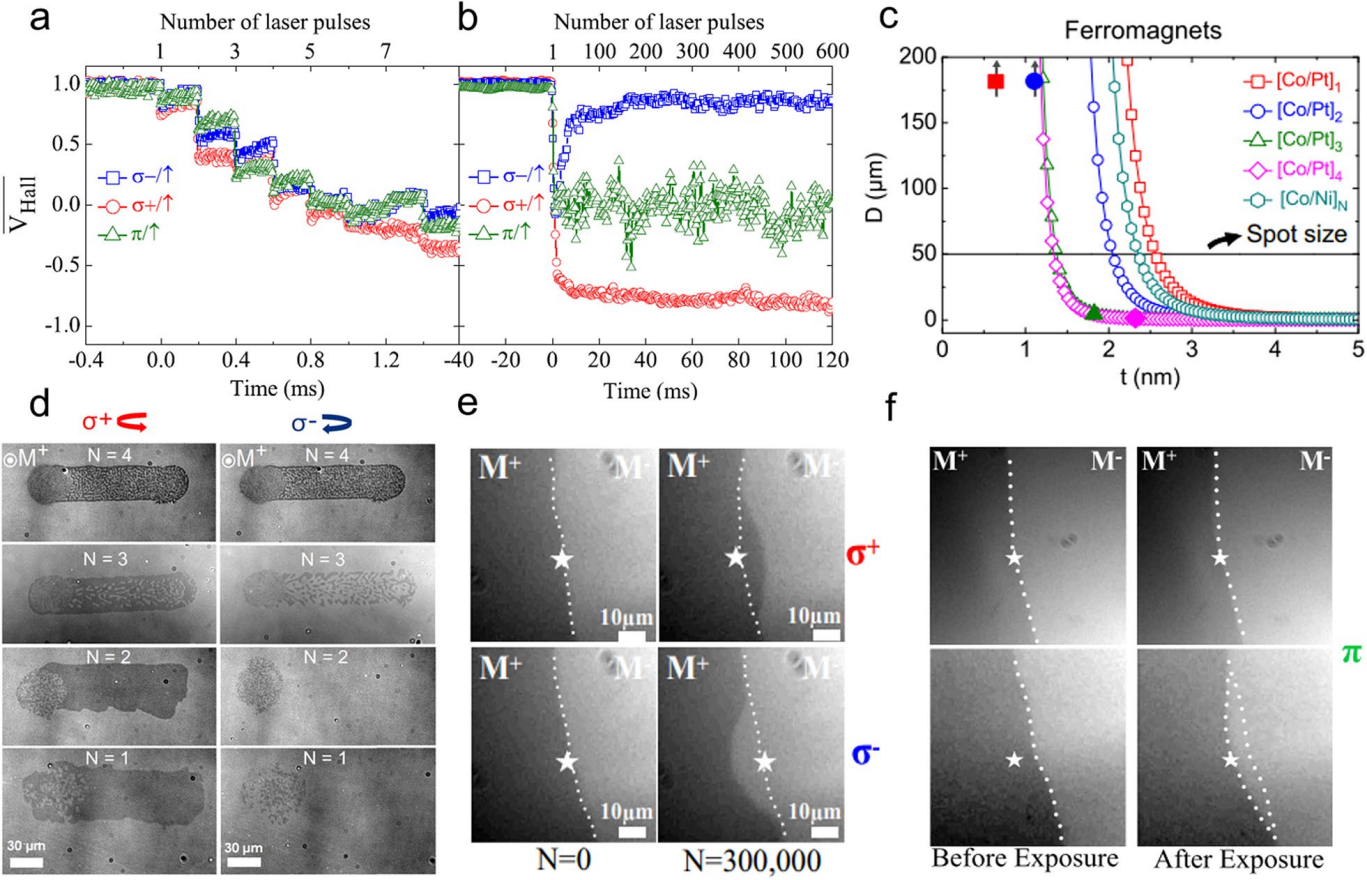
Evolution of the anomalous voltage of Pt/Co/Pt at **a** fine and **b** broad timescales. **c** AO-HDS results of Co/Pt multilayers with different repeats. **d** Calculated magnetic domain size versus the magnetic thickness of Co/Pt multilayers. Faraday image of Pt/Co/Pt sample with an incident of **e** circularly (${\sigma }^{+}$ and (${\sigma }^{-}$) or **f** linearly (π) polarized laser. The white star symbol indicates the center of the laser spot. Figure **a** and **b** are reproduced with permission from ref. [96]. Copyright (2016) American Physical Society. Figure **c** and **d** are reproduced with permission from ref. [99]. Copyright (2016) American Physical Society. Figure **e** and **f** are reproduced with permission from ref. [103]. Copyright (2018) American Physical Society

TR-MOKE usually includes a delay line with timescales ranging from femtosecond to tens of nanoseconds at most. To overcome this limitation, we can utilize electrical characterization to reach microsecond and beyond. Using this approach, El Hadri et al*.* revealed the cumulative characteristic of AO-HDS of ferromagnetic materials [95, 96]. The perpendicular magnetized Pt/Co/Pt is patterned into a Hall bar structure and the magnetization state is quantified by Hall voltage based on anomalous Hall effect [96]. Figure 5a and b show the time evolution of Hall voltage in fine and broad timescales under the illumination of a pulsed laser with a 5 kHz repetition rate. In the first eight pulses, the Hall voltage monotonically decreases and reaches zero voltage regardless of the polarization of the incident laser (Fig. 5a). Subsequently, the magnitudes of the voltage in the three cases diverge with multiple pulses (Fig. 5b). The ultimate Hall voltage states agree with the AO-HDS feature [16]. The cumulative magnetic switching behavior is also found in the granular ferromagnetic FePt [97, 98], which is a potential high-density magnetic recording material. The demagnetization multidomain after a few pulses and magnetization reversal after multiple pulses indicate the role of the magnetic domain. Hadri et al*.* investigated AO-HDS of [Co/Pt]_N_ with different repeats of the Co/Pt layer [99]. As seen in Fig. 5d, AO-HDS only exists when N = 1 and 2. One can calculate the expected domain size at different thicknesses [100]. The result is depicted in Fig. 5c. The conclusion is that AO-HDS happens when the domain size exceeds the size of the laser spot. Other experimental results also imply the importance of the intrinsic domain size in comparison to the laser spot size [101].

It is generally accepted that AO-HDS of ferromagnetic materials involves two steps. The first step is the helicity-independent demagnetization by laser heating and the nucleation of reversal domains. Then a helicity-dependent domain wall motion preferably expands the reversed domains [102–104]. When a laser impinges on the center of a domain wall, depending on the helicity of laser, the domain moves out of the hottest area distinctly [103] (Fig. 5e). The domain wall only moves when the laser fluence is above a certain value, indicating the domain wall pinning. Next, linearly polarized light could be adopted to clarify the role of laser heating (Fig. 5f). When the laser spot is at the center of the domain wall, there is no displacement, because the temperature profile is symmetrical with respect to the domain wall. In contrast, the domain wall moves toward the hottest area when the laser spot is away from the domain wall. Therefore, the domain wall pinning, laser heating, and light helicity contribute together to the domain wall motion in ferromagnetic materials.

**Fig. 6:**
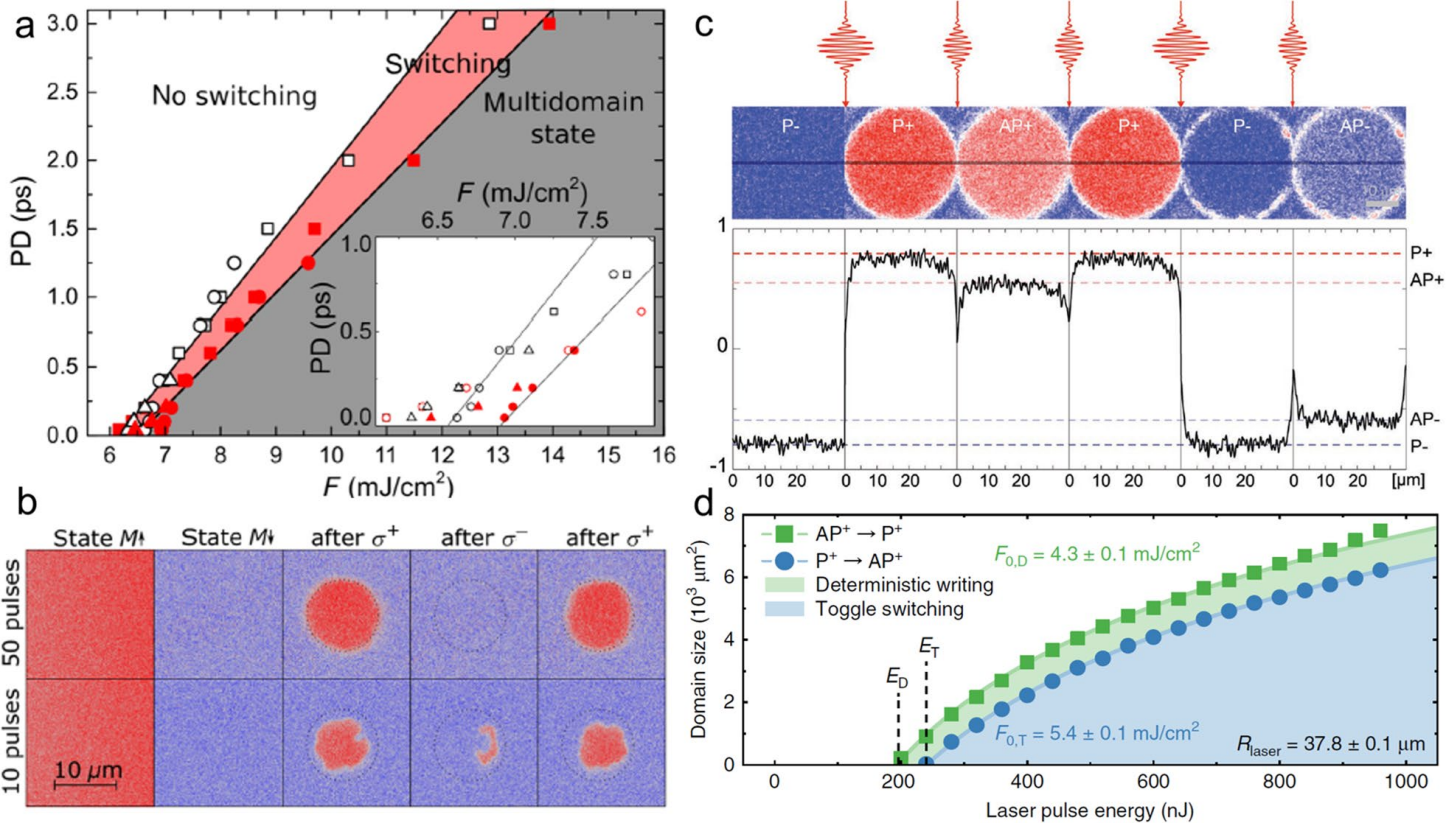
**a** Magnetization diagram with respect to the fluence, pulse duration (PD), and laser beam diameter. **b** Magneto-optical Kerr imaging after 50 and 10 pulses with laser duration of 2 ps. **c** All-optical switching of GdFeCo/Cu/[Co/Pt] spin-valve structure. **d** Dependence of measured switched domain size with pump laser pulse energy. Figure **a** and **b** are reproduced with permission from ref. [105]. Copyright (2019) American Physical Society. Figure **c** is reproduced with permission from ref. [108]. Copyright (2018) WILEY‐VCH Verlag GmbH & Co. KGaA, Weinheim. Figure **d** is reproduced with permission from ref. [109], under a Creative Commons Attribution 4.0 International License

The AO-HDS of ferromagnetic materials is sensitive to laser parameters such as the fluence, pulse duration, repetition rate, and beam size [102, 103]. Meanwhile, it is generally a cumulative process, meaning that multiple pulses are needed. This characteristic is not favorable for ultrafast and efficient magnetic bit writing. Therefore, efforts have been devoted towards single-shot AO-HDS of ferromagnetic materials [101, 105–108]. A representative magnetic state diagram for Co/Pt stack as a function of laser fluence, pulse duration, and beam diameter is depicted in Fig. 6a [105]. It is found that the longer laser duration is preferred for the efficient switching of magnetization. With optimized laser parameters, the number of required pulses for complete AO-HDS can be reduced to 50 (Fig. 6b). Even 10 pulses can give rise to a reversal of magnetization with considerable contrast. Considering the two-step feature of AO-HDS of ferromagnetic materials, Yamada et al*.* have proposed and demonstrated a method of using dual laser pulses to achieve efficient AO-HDS in a single stack of Pt/Co/Pt thin film. In the proposed scheme, the first linearly polarized pulse with a duration of 90 fs promotes the stochastic nucleation of the reversal domain, and the subsequent circularly polarized pulse with a duration of 3 ps drives the domain wall motion [106]. The required number of pulses can be further reduced to 4 pairs of dual pulses. Another way to promote the efficient AO-HDS of ferromagnetic materials is to take advantage of ferrimagnets and its spin current diffusion [107–109]. Specifically, a single 35 fs laser can determinately switch the GeFeCo/Cu/Co/Pt spin-valve structure [108], in which Cu serves as the spacing layer. Both GeFeCo and Co/Pt layers are initially magnetized along the negative magnetic field direction (P− state). After a relatively strong single pulse irradiation, both layers are switched along positive magnetic field direction (P+ state), as shown in Fig. 6c. However, using a relatively weak single pulse can only switch the top GdFeCo layer (AP+ state). The final state of Co/Pt is dependent on the magnetization state of the GdFeCo layer, indicating the interlayer spin angular momentum transfer. The role of spin angular momentum transfer is further verified by a control experiment with the insertion of a thin layer of Pt, where the Pt layer cut off the spin current transport and no switching of Co/Pt layer is observed (see Fig. 5 in ref. [108]). In the meantime, the non-local transfer of spin angular momentum from ferromagnet Co/Ni can deterministically switch the magnetization of ferrimagnet Co/Gd by a single pulse, in sharp contrast to a typical toggle switching observed in ferrimagnets. Figure 6d summarizes the dependence of the switched domain size on the laser pulse energy for a sample with an initial state of either AP^+^ or P^+^ state. The switching of AP^+^ to P^+^ requires a lower threshold (E_D_) that the threshold (E_T_) for the switching of P^+^ to AP^+^. The reason for this dissymmetry is that the polarization of generated spin current from Co/Ni is parallel (antiparallel) to Co/Gd in P^+^ (AP^+^) state, thus hindering (assisting) the magnetic switching of Co/Gd. The breaking of the symmetry of toggle switching results in the deterministic switching of Co/Ni as indicated by the green region in Fig. 6d.

**Fig. 7:**
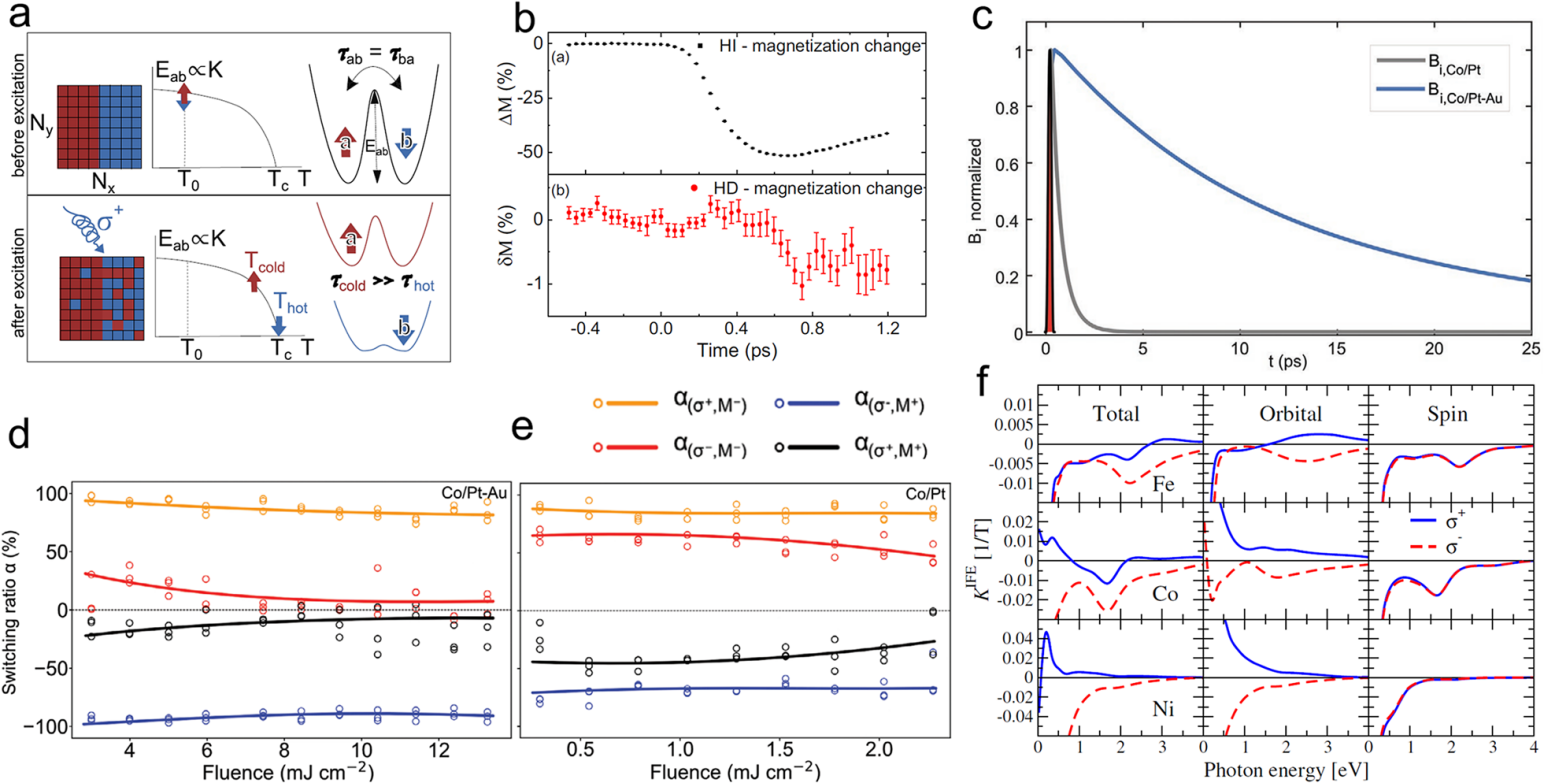
**a** Graphic representation of the AO-HDS model in ferromagnets [111]. **b** Helicity-independent and helicity-dependent magnetization change extracted from TR-MOKE results [113]. **c** Simulated effective magnetic field for CoPt/Au and CoPt samples with a laser pulse duration of 200 fs. Pulse-dependent switching ratio for **d** CoPt/Au and **e** CoPt samples. **f** Calculated photon energy and laser polarization-dependent total, orbital, and spin IFE constant K^IFE^. Figure **a** is reproduced with permission from ref. [111]. Copyright (2016) American Physical Society. Figure **b** is reproduced with permission from ref. [113]. Copyright (2019) American Physical Society. Figure **c, d**, and **e** are reproduced with permission from ref. [116], Copyright (2020) WILEY‐VCH Verlag GmbH & Co. KGaA, Weinheim. Figure **f** is reproduced with permission from ref. [123]. Copyright (2016) American Physical Society

The mechanisms for degeneracy breaking in AO-HDS of ferromagnetic materials are still inconclusive [110]. Two commonly accepted mechanisms are thermal magnetic circular dichroism (MCD) effect [111–113] and non-thermal IFE effect [98, 114, 115]. In 2016, Gorchon et al*.* proposed a microscopic model to describe the multi-shot AO-HDS in ferromagnets based on MCD [111]. Intuitively, the model states that an ultrafast circularly polarized pulse would heat the material to the vicinity of T_c_. Because of MCD, the different magnetized areas will absorb a different amount of heat, resulting in hotter and cooler regions. The hotter region has a lower energy barrier between the up and down state, giving rise to different switching possibilities (Fig. 7a). Therefore, deterministic switching takes place after multiple pulses. A similar model was proposed for granular ferromagnet FePt at the same time [112]. Both works claim that MCD value as small as 2% could lead to AO-HDS in ferromagnets. As mentioned earlier, the helicity-dependent domain wall displacement is believed to be an underlying mechanism and only a small amount of laser fluence difference will significantly affect the domain wall motion. However, the MCD signal has to be determined to compare with experiments, while so far only a few experimental works have been reported [113, 118]. Typical TR-MOKE spectroscopy was used to measure the helicity-dependent magnetization dynamics in CoTb alloy for four combinations of light helicity and initial magnetization direction [113]. Although the experiment was done on ferrimagnet, the MCD mechanism is also applicable to ferromagnets. The extrapolated temporal helicity-independent and helicity-dependent part magnetization changes are summarized in Fig. 7b. The helicity-independent part exhibits a typical 50% demagnetization curve with τ_M_ ≈ 500 fs. In contrast, helicity-dependent part suggests an about 0.8%-1% percent demagnetization, which is consistent with the typical expected MCD value. In particular, it determines that the MCD sign is negative, in agreement with domain wall displacement experiments (see Fig. 4 in ref. [114]).

The IFE has been considered as the driving force since the first discovery of the AO-HDS in GdFeCo [14]. In the simulation of AO-HDS in ferromagnets, the IFE is treated as an effective magnetic field based on the modified three temperature model [114]. The result shows that the switching requires a minimal 0.15 ps duration of IFE field with an extreme value of around 20 T. Similar results were also found in FePt [112, 115]. Recently, we found that the generated effective magnetic field can be prolonged in Co/Pt-Au heterostructures [116, 117]. Based on the classic Lenz’s law, the decrease of the IFE-induced magnetic field wakes a loop current in the Au top-cladding layer. It counteracts the decrease of the magnetic flux, and hence prolongs the effective magnetic field. Figure 7c plots the simulated effective fields for a Co/Pt-Au heterostructure and also a bare Co/Pt thin film. Since Au has a larger electrical conductivity compared to the Co/Pt film, the effective magnetic field decays very slowly and lasts much longer than the 200-fs laser pulse. As a result, we can achieve pronounced and robust AO-HDS in the Co/Pt-Au structure. We experimentally compared the AO-HDS results of Co/Pt samples with and without the Au cladding layer. Helicity-dependent deterministic switching in the Co/Pt-Au sample happens across a wide range of laser fluences (Fig. 7d), while we only observe weak helicity-dependent switching in the bare Co/Pt thin film (Fig. 7e). Furthermore, the Au layer serves as a good heat sink, which also facilitates the process of AO-HDS.

The fundamental origin of IFE in metals is still unclear [119–122]. A recent first-principle calculation offered a new insight into IFE [123]. The calculation was based on materials specific electronic structure, including the light absorption of metals that had not been considered before. Contrary to previously assumed antisymmetric induced magnetization concerning light helicity, the laser-induced magnetization is nonsymmetric for ferromagnets while it is still antisymmetric for non-magnetic metals (Fig. 7f). The induced orbital and spin IFE are comparable in 3*d* ferromagnets. The spin IFE is not antisymmetric, and even its magnitude does not change with light helicity. Quantitatively, the calculated effective opto-magnetic field is about one hundred Tesla for Co. This theory was later applied to study the magnetization switching in FePt nanoparticles and produced consistent results with experimental observations [98]. The recent observation of large light-induced magnetic moment in plasmonic gold nanoparticles also supports the IFE theory [124].

## Conclusion and perspective

In this review, we have summarized the recent studies of laser-induced ultrafast magnetization dynamics, particularly in metallic ferromagnetic materials. The ultrashort laser pulses excite metallic ferromagnetic materials into a highly thermalized non-equilibrium state in which magnetic states could be significantly modified in an ultrafast way through light-matter interactions. It has triggered fundamental questions as to what the origin of ultrafast demagnetization is and how we can properly model this process since the conventional thermodynamic description of magnetization is not applicable in this case. Different local spin-flip scattering mechanisms were firstly considered and fitted well with experimental results. However, they cannot predict the laser-induced magnetization dynamics. Alternatively, the laser-induced spin current could also explain the ultrafast demagnetization. Taking the contribution of superdiffusive hot electrons into account in rt-TDDFT simulation might give a complete picture of ultrafast demagnetization in the future. On the other hand, ASD and LLB simulation provide distinct techniques to model the ultrafast magnetization dynamics. However, the flow of angular momentum has not been systematically considered, which calls for further investigation.

Emphasis should be drawn on the laser-induced spin current. Advanced characterization techniques have identified the behavior of spin current inside metallic thin films, but the quantitative description of laser-induced spin current is still lacking. The estimated induced spin current density is up to 10^12^–10^13^ A/cm^2^ [53, 54, 57], larger than the commonly used density in current-drive magnetic devices [125]. Therefore, it provides a potential ultrafast method to control the magnetization dynamics and the switching of ferromagnetic materials by STT. In the meantime, it has been proposed as a superior candidate among conventional terahertz sources [126]. For example, spin injection into two-dimensional materials with giant terahertz emission has been recently reported [127].

It is still early to say that the ultrafast AOS of ferromagnetic materials will be a solution to the current bottleneck in the magnetic recording industry. One reason is that the cumulative switching of ferromagnetic materials prevents efficient magnetic bit writing. Fortunately, the two-stages switching was well identified, allowing us to optimize material and control parameters towards single-pulse magnetization reversal [105–108]. Only 4 pulse pairs could completely switch a Pt/Co/Pt stack using the dual-pulse approach [106]. Taking advantage of laser-induced contributions, such as harnessing spin-polarized hot electrons [107, 108] and enhancing opto-magnetic effect [116], could help us to further push AO-HDS of ferromagnetic materials. On the other hand, the cumulative AOS may benefit the design of neuromorphic architectures [128, 129]. To achieve a high-density recording, AOS of nanoscale magnetic nanostructures instead of continuous media should be considered. Up to now, only a few experimental works on AOS in nanostructures have been reported [9, 16, 97, 98], because of the challenges in characterizing and controlling a magnetic bit with considerably small spatial resolution. Plasmonic effect is expected to be able to control magnetic data bits [130–132] at the nanoscale, and using magnetic tunnel junctions would be a simple solution for detection [7].

In conclusion, all-optical switching phenomena in ferromagnetic materials have opened a new paradigm in science and engineering. Further development in this emerging field will benefit from the synergetic effort of researchers from different disciplines, including materials science, physics, optics, and nanophotonics. The research findings will potentially transform data storage technologies with unprecedented speed to meet the demands of the big data era.

## Competing interests

The authors declare that they have no competing interests.

## Data Availability

Not applicable.

## Abbreviations

3TM: Three-temperature model
AOS: All-optical switching
AO-HDS: All-optical helicity-dependent switching
ASD: Atomistic spin dynamics
CMOS: Complementary metal-oxide-semiconductor
DFT: Density function theory
EY: Elliott–Yafet
HD: Helicity-dependent
HI: Helicity-independent
IFE: Inverse Faraday effect
LLB: Landau–Lifshitz–Bloch
LLG: Landau–Lifshitz–Gilbert
MCD: Magnetic circular dichroism
MRAM: Magnetic random-access memory
OSTT: Optical spin-transfer torque
rt-TDDFT: Real-time time-dependent density functional theory
SDSE: Spin-dependent Seebeck effect
SHG: Second harmonic generation
SOC: Spin–orbit coupling
SOT: Spin–orbit torque
STT: Spin-transfer torque
TR-MOKE: Time-resolved magneto-optical Kerr effect
XUV: Extreme-ultraviolet
